# Neglected Needs of Family Caregivers during the COVID-19 Pandemic and What They Need Now: A Qualitative Study

**DOI:** 10.3390/diseases9040070

**Published:** 2021-10-13

**Authors:** Jasneet Parmar, Sharon Anderson, Bonnie Dobbs, Peter George J. Tian, Lesley Charles, Jean Triscott, Jennifer Stickney-Lee, Suzette Brémault-Phillips, Sandy Sereda, Lisa Poole

**Affiliations:** 1 Division of Care of the Elderly, Department of Family Medicine, University of Alberta, Edmonton, AB T6G 2T4, Canada; Jasneet.parmar@albertahealthservices.ca (J.P.); bdobbs@ualberta.ca (B.D.); peter.tian@ualberta.ca (P.G.J.T.); lesley.charles@albertahealthservices.ca (L.C.); Jean.Triscott@albertahealthservices.ca (J.T.); Jennifer.stickneylee@albertahealthservices.ca (J.S.-L.); 2 Home Living Edmonton Zone, Alberta Health Services, Edmonton, AB T6G 2G4, Canada; 3 Medically At-Risk Driver Centre, University of Alberta, Edmonton, AB T6G 2T4, Canada; 4 Department of Occupational Therapy, University of Alberta, Edmonton, AB T6G 2G4, Canada; suzette.bremault-phillips@ualberta.ca; 5 Caregivers Alberta, Edmonton, AB T5B 1R1, Canada; ssereda@caregiversalberta.ca; 6 Dementia Advocacy Canada, Dementia Network Calgary’s Strategic Council, Gordie Howe C.A.R.E.S. & the Brenda Strafford Foundation Dementia Friendly Communities, Calgary, AB T3B 0K7, Canada; llpoole@me.com

**Keywords:** family caregivers, informal caregivers, unpaid caregivers, COVID-19, navigation, caregiving trajectory, interpretive description, continuing care

## Abstract

COVID-19 has had a negative impact on family caregivers, whether the care receivers lived with the caregiver, in a separate community home, in supportive living, or in long-term care. This qualitative study examines the points of view of family caregivers who care in diverse settings. Family caregivers were asked to describe what could have been done to support them during the COVID-19 pandemic and to suggest supports they need in the future as the pandemic wanes. Thorne’s interpretive qualitative methodology was employed to examine current caregiver concerns. Thirty-two family caregivers participated. Family caregivers thought the under-resourced, continuing care system delayed pandemic planning, and that silos in health and community systems made caregiving more difficult. Family caregivers want their roles to be recognized in policy, and they cite the need for improvements in communication and navigation. The growth in demand for family caregivers and their contributions to the healthcare system make it critical that the family caregiver role be recognized in policy, funding, and practice.

## 1. Introduction

The COVID-19 pandemic exposed weaknesses in Canadian healthcare systems; however, the pandemic might provide an impetus for system redesign [1,2]. Support for family caregivers is at a dangerous low. Illustratively, in his 2016 review of the textbook *Supporting Families and Carers: A Nursing Perspective,* Dr. Colin Young submitted that “working with, and providing support to, carers seems to have gone off the political and policy radar” [3]. Before the COVID-19 pandemic began, family caregivers provided 75–90% of the care to people needing care in the community [4,5] and assisted with 10–30% of the care in congregate living settings [6]. Yet family caregivers were marginalized by healthcare systems and procedures [7,8,9]. Recent reviews also recognize that effective support interventions for family caregivers exist but are rarely translated into widespread practice [4,8,10,11]. These reviewers stressed that, with a rapidly growing population of older adults, effective caregiver supports must be developed to deal with the higher demands that family caregivers are expected to fulfil.

Worldwide, family caregivers were at higher risk of social isolation, stress, neglected health needs, and loss of services and supports during the COVID-19 pandemic [12,13,14,15,16,17]. Family caregivers caring for people living in community homes were caring for many more hours a week than they were before the COVID-19 pandemic [12,13,14,15]. Patients were discharged early from acute care and rehabilitation [18,19]. In many communities, social and physical activities, support groups, and day programs closed. Home care and respite services were reduced [20,21]. In many Canadian provinces, when awareness of the pandemic dawned around February/March 2020, the family caregivers of congregate living residents (e.g., supportive or assisted living, long-term care) were restricted from entering the buildings in which congregate living residents reside [14,22]. “Window”, outdoor, or virtual visits replaced visits that entailed physical contact. In July or August 2020, essential family caregivers equipped with personal protective equipment were allowed to enter a resident’s room. Loneliness, anxiety, and a sense of burden rose dramatically for family caregivers, regardless of the care setting [14,23,24,25].

While it was and continues to be important to capture the ongoing impact of the COVID-19 pandemic on family caregivers, the aim of this qualitative study is to understand what family caregivers thought could have been done to support them as the COVID-19 pandemic continued, and what supports they feel they need in the future. First, we briefly review the impacts that COVID-19 had on family caregivers.

Impacts of the COVID-19 Pandemic on Family Caregivers

The COVID-19 pandemic and the public health protocols to control and respond to the disease added to family caregiver work and stress [15,26,27,28]. Health and social care services were reduced. Hospitals discharged patients sooner and suspended non-emergency services and outpatient clinics. Family caregiver and patient support groups and day and respite care closed. Family caregivers were locked out of hospitals, supportive/assisted living, and long-term care [29,30,31]. The complexity of care and the weekly hours of care work increased substantially for family caregivers caring in community homes [12,13,17,32]. In the Canadian province of Alberta, family caregivers were restricted from entering supportive living and long-term care from March 2020 until July 27, 2020 [20,22]. However, family caregiver restrictions to entering congregate care settings changed with wave two and wave three of the COVID-19 pandemic and depended on the facility. Anxiety, burden, and loneliness increased [25,33,34,35,36]. Family caregivers’ perspectives on what they thought would have helped them and what supports are needed to support family caregivers now and in the future are reported in the Results section.

## 2. Materials and Methods

This paper reports the findings of follow-up interviews with a selection of family caregivers who had completed an online survey in July 2020 entitled “Impacts of COVID-19 on Alberta Family Caregivers”. The survey results are reported elsewhere [14,37]. The interviews addressed two research questions not answered in the survey:

1. What do family caregivers think could have been done to support them during the COVID-19 pandemic?
2. What do family caregivers think they need to assist them to care for others and to maintain their own wellbeing as the risk of COVID-19 decreases?

Family caregivers and their caregiving situations are diverse. Family caregivers’ needs are also diverse and change throughout the care trajectory. We used Thorne’s [38] qualitative interpretive description methodology to capture the complexity of the situation, while acknowledging that multiple interpretations exist. The approach was designed to seek an in-depth understanding of the family caregiver situation and to suggest results that will inform practice and policy [38,39]. Ethics approval for this study was granted by the Health Research Ethics Board (Pro00097996) at the University of Alberta.

### 2.1. Context

Alberta is one of 13 provinces and territories in Canada. It is a land-locked, western prairie province with a population of 4,371,316. To date, Alberta has experienced three waves of COVID-19 exacerbations. There were 19 cases of COVID-19 in Alberta on 11 March 2020, when the World Health Organization declared the pandemic [40]. Public health facility protocols dictated that home and community care programs reduced face-to-face services and congregate healthcare services restricted entry to healthcare providers on 11 March 2020 (wave 1). Congregate care restrictions began to ease on 13 July 2020. Hospitals, supportive living, and long-term care facilities began to allow access to one or two designated family caregivers to support patients/residents [7,9]. Home care resumed services as needs and COVID-19 public health protocols dictated. Family caregivers of congregate care residents experienced significant variation in the access to care receivers, as admission depended on facility discretion and capacity to accommodate family caregivers. COVID-19 cases began to increase in October 2020. The Alberta Chief Medical Officer of Health declared a second COVID-19 wave on 16 November 2020. Restrictions were relaxed on 29 January 2021, as case counts decreased. By 30 March 2021, Alberta was experiencing a third wave of COVID-19 and encountered the highest infection positivity rate in Canada.

### 2.2. Sample Selection

Consistent with interpretive description methodology, we used convenience sampling [41,42]. To ensure representative credibility, we interviewed family caregivers who had completed the survey and had consented to further interviews (578/604). We separated those who consented to participate in interviews by receiver’s residence (same home, separate home, supportive living, long-term care), and then sent email invitations to every fifth caregiver. Participants who replied to the email invitation were emailed or mailed information about the study, and the Research Coordinator organized a mutually agreeable time for the interview. Interviews were held virtually on ZOOM or by telephone. We received Health Ethics Research Board approval to obtain verbal consent from participants (Pro00097996).

### 2.3. Data Collection

We began the family caregiver interviews at a time when people were hopeful that the COVID-19 pandemic might be controlled with vaccines [43]. The second COVID-19 wave was diminishing, and many of the long-term care and supportive living residents had received their first COVID-19 vaccination. We collected data from 15 January to 15 April 2021, guided by a semi-structured interview guide (see Supplementary Material 1: Interview Guide). Interviews were carried out by a trained (PhD) research coordinator who had experience conducting qualitative interviews [44]. Participants were given the choice of being interviewed on ZOOM or by telephone. Verbal consent was obtained from each participant for the interview and for the audio-recording of the interview. Interviews were digitally recorded and averaged one hour in length (45–75 min).

### 2.4. Data Analysis

Data analysis proceeded concurrently with data collection [41]. Interviews were transcribed verbatim, and identifying information was removed. We analyzed the data thematically, as suggested by Thorne [41]. Thematic analysis is a flexible qualitative method used to explore the different perspectives held by research participants; it highlights the similarities and divergences in their viewpoints, and generates thematic insights [45,46]. We methodically followed Braun and Clarke’s [45,46] six stages of analysis (see Supplementary Material Table S1: Stages of Thematic Analysis).

To become familiar with the data and to generate first impressions of meaning (stage one), two members of the research team independently read participants’ qualitative responses. They made notes of their impressions on MS Word transcripts. They discussed the initial impressions, then imported the data into NVivo. In stage two, members of the research team worked separately to inductively generate initial open codes. In stage three, team members worked together to generate categories. Patterns within the open codes were identified and codes with similar attributes and meanings were grouped. The categories were then refined into preliminary themes (stage four). At stage four, we discussed how the knowledge might apply in family caregiving and clinical practice, and how this knowledge might have made life easier in the pandemic. We now had family caregivers’ perceptions of what they needed in the future. We then reread the transcripts to name and confirm the final themes (stage five). The report was generated (stage six) and discussed at a final team meeting.

## 3. Results

A total of 32 family caregivers took part in this research. The caregiving took place in four different situations: the care receiver lived with the caregiver (*n* = 10); the care receiver lived in a community home, separate from the caregiver (*n* = 7); the care receiver lived in a supportive living residence (*n* = 5); and the care receiver lived in a long-term care residence (*n* = 10). Three family caregivers cared for more than one person. Care receivers included a mother or father (*n* = 14), husband or wife (*n* = 8), a child under 18 (*n* = 4), grandparent (*n* = 4), adult son or daughter (*n* = 2), and aunts (*n* = 2). Family caregivers were caring for Albertans with one or more conditions including dementia, frailty, end-of-life care, Parkinson’s disease, multiple system atrophy, autism, fetal alcohol syndrome, vision deficiency, hearing disability, or organ transplant.

We began the interviews by asking family caregivers about their care situation. We then asked them what concerned them most about the COVID-19 pandemic right now, then turned to what they thought might have helped them in the pandemic so far, and what they thought they needed as family caregivers in the future, based on their experiences in the pandemic and what they had learned. We present the results in this order.

### 3.1. Family Caregivers’ Situations and Their Main Concerns

In general, talking about the care receivers’ needs interested the family caregivers more than talking about their own needs. Despite their diverse situations at the time of the interviews, most participants wanted supports that balanced the risk of COVID-19 infection with the receivers’ needs for emotional, affirmational, and practical support. Most participants thought that family caregivers could provide face-to-face care safely. They gave three arguments for the view. (1) Caregivers contended that family caregivers were far less likely to spread the COVID-19 infection than support staff at care venues. (2) Family caregivers of both children and adults tied isolation and lack of social interaction to the care receiver’s deteriorating cognitive and physical health and to their responsive behaviors. Family caregivers of older people who were vaccinated wondered why the care receiver’s quality of life was not prioritized. (3) Family caregivers pointed out that they were the most knowledgeable about the care receivers and that they should be considered as partners in care and decision making. Some even reported that with their experience as a family caregiver, they enacted COVID-19 prevention protocols in advance of public health protocols.

Family caregivers contended that the time lost due to the COVID-19 pandemic had been “stolen”. A few of the caregivers interviewed thought it was paramount to ensure that care receivers were not exposed to the COVID-19 virus. Two caregivers (one in supportive living; one in long-term care) worried that “other caregivers” or “visitors” would spread the virus even though most of the residents were vaccinated. Table 1 displays the themed exemplar quotes from the four care settings placed side by side to show the theme concordance across settings.

### 3.2. What Could Have Been Done to Support Family Caregivers during the Pandemic?

The conversation about family caregivers’ greatest concerns led to a discussion about what might have been done differently to support family caregivers to maintain their own wellbeing. There were three main themes: (a) an under-resourced continuing care system, (b) delayed pandemic plan development, and (c) the increased presence of silos in health systems which blocked communication between healthcare providers and services (e.g., emergency departments, primary care practices, home care).

#### 3.2.1. Increase Resources to the Continuing Care System

Family caregivers noted that the COVID-19 pandemic exposed an already stressed, underfunded continuing care system. Here, “continuing care” refers to community and home care services, supportive and assisted living, group homes, and long-term care. Family caregivers noted that, even when home care services were engaged, the caregivers were still doing most of the care work. Family caregivers reported that home care services—such as respite or assistance with personal care (e.g., showering)—did help them to sustain care, but they thought that more resources could have been allocated to home care services.

Family caregivers in group homes, in supportive living, and in long-term care spoke about understaffing prior to the pandemic. They noted that many healthcare staff worked part-time in more than one setting (home care, hospital, supportive living, long-term care) due to lack of funding. Several caregivers reported that they spent considerable time supplementing resident care because they thought it assisted the staff; it also enabled them to spend time with residents who did not have family caregivers. They felt the extra time spent enabled their care receiver to thrive.

Family caregivers emphasized that during the COVID-19 pandemic, home care and congregate care employees did their best to provide good physical care despite the short time they were able to spend with the care recipient. Even in care settings where there had been COVID-19 outbreaks, family caregivers complimented the dedication they observed in staff. However, they pointed out that staff had to focus on basic care tasks rather than the cognitive, emotional, and relational needs of care receivers because they had a lot to do in too little time. Family caregivers thought that more resources should have been allocated to home care and congregate care staffing.

#### 3.2.2. Develop a Timelier Pandemic Plan

Family caregivers thought the initial closures of community services and the restricted entry to supportive living and long-term care were reasonable because everyone was uncertain about how to manage the COVID-19 virus. However, they thought that protocols to support the efforts and the health of family caregivers should have been developed much more quickly. Family caregivers caring in community homes sensed their caregiving was invisible or taken for granted. Many believed that support and respite programs (e.g., fitness facilities for people with disabilities, adult day programs) could have resumed safely. Family caregivers who provided supportive living and long-term care felt their caregiving role was unappreciated. The initial “visitor” designation excluded them from the building and devalued their work. When one or two family caregivers were designated as being essential to the care recipients in hospitals, supportive living, and long-term care facilities, the staff-family-caregiver relationship remained paternalistic. For the most part, family caregivers felt they were told what to do, tolerated rather than welcomed.

Healthcare and community organizations turned to phone calls and the Internet to communicate. Email, videotelephony such as Facetime or Google Meet, or online meeting applications were often used for communication. Initially, communication was mainly about restrictions, closures, and organizational responses to the public health restrictions. Family caregivers noted that the new communication tools such as ZOOM meetings or email newsletters provided them with useful information about what the organization was doing to cope with the pandemic. They enjoyed the flexibility of online meetings with healthcare providers and other family caregivers and with condition-specific (e.g., autism, Alzheimer’s Parkinson’s) organizations, but missed physical social interactions. Family caregivers appreciated most of the communication inherent in congregate care settings. Moving family council meetings online enabled more family caregivers to attend. However, family caregivers thought communications about residents’ emotional and physical wellbeing could have been improved. Over three quarters of the family caregivers reported that having been told the resident was “fine”, they were shocked by the cognitive and physical deterioration of the resident when they were allowed a face-to-face visit.

#### 3.2.3. Reduce Silos in Healthcare

Family caregivers felt there was a need to coordinate services across professionals within teams and between care settings because COVID-19 exacerbated silos (divisive mindsets and practices that block communication between healthcare services [47,48]). Family caregivers reported they had difficulty finding services and supports that met their needs. For example, one caregiver reported that although she had been caring for over 10 years, she just recently discovered the provincial caregivers’ association.

COVID-19 exacerbated the difficulty in navigating the healthcare system. The volume of online information grew exponentially during the pandemic, making it more difficult to find assistance. When assistance was found, it was difficult the obtain from healthcare and social care providers who were working from home. The online forms were complex and difficult to complete, and sometimes required documents to be attached to the request. Appointments and services were cancelled, or their availability was limited. Family caregivers thought there was more miscommunication in handoffs between professionals because family caregivers were not asked for their knowledge about the care-receiver or excluded from consultations. To illustrate, a family caregiver reported that a palliative care physician thought he was treating a cancer diagnosis obtained from outdated, incomplete records when the condition was heart failure.

Family caregivers struggled to understand the levels of care in the congregate care system. They said it took time to understand exactly what care was included in the contracts and fees. They needed to understand how much of the care they were responsible for, what care they would have to hire another provider to do, and what care they were allowed to assist with. COVID-19 exacerbated the silos between formal staff and family caregivers.

### 3.3. What Supports do Family Caregivers Think They Need in the Future Based on What They Learned in the COVID-19 Pandemic?

Interview participants were hopeful that increased awareness of family caregivers created in COVID-19 would offer opportunities to improve the community and healthcare systems in the future. The improvements they anticipated included: (a) recognition of the caregiver role; (b) improvements in partnering communications with health providers; and (c) assistance for caregivers to navigate community, healthcare, and continuing care systems.

#### 3.3.1. Recognition of the Family Caregiver Role

The participants contended that the family caregiver role needed to be recognized in policy and in practice. They wanted to be consulted in discussions about policies and practices that affected them and the people they cared for. They thought that during the COVID-19 pandemic, policy makers and health system leaders were focusing on the protection of the health system and were not considering the impacts the pandemic had on family caregivers. They noted that because family caregivers care in the community, they were essential to supporting the overstretched health systems, yet top-down decisions were made without including input from family caregivers or patients/residents.

Family caregivers of congregate care residents argued that current policies positioned them as peripheral to congregate care functioning. They asserted that referring to family caregivers as “visitors” marginalized their work, and that essential family caregiver policies did not recognize family caregivers as partners in healthcare. To emphasize the family caregivers’ inferior positioning, one family caregiver reported that she was not even allowed to drink a glass of water, even though she was spending a full eight-hour day providing care.

#### 3.3.2. Partnering with Communication between Healthcare Providers and Family Caregivers

Family caregivers wanted their knowledge of the care receiver and the care receiver’s needs to be considered in conversations with healthcare providers (staff). When the COVID-19 pandemic was initially declared, family caregivers reported communications to them were primarily about program and healthcare closures and restrictions of family caregivers. Then, healthcare and community organizations turned to phone calls, Internet communications (emails), and videotelephony such as Facetime or Google Meet, or online meeting applications.

Generally, family caregivers appreciated the flexibility and access these tools. Not having to drive to appointments, pay for parking, and wait in a crowded reception room were particularly attractive outcomes. However, family caregivers wanted to be able to email their monitoring results (e.g., blood pressure, blood sugar) to healthcare providers, but several reported still having to fax or drop off a paper copy. Family caregivers with access to the Internet and computers found it easy to access online support. Rural family caregivers wanted Internet service improved. Rural and urban family caregivers reported that phone or Internet communication did not work well with care receivers who had cognitive, hearing, or vision impairments.

Family caregivers noted that the communication content was more about the operations of the organization and routines to cope with the pandemic than about the needs of the person they cared for, or family caregivers’ needs. They wanted information specifically about the care recipient or to their needs, information that could aid in their care management and decision making. Family caregivers reported they were rarely asked about their needs. In fact, several caregivers burst into tears when asked.

#### 3.3.3. Access to Resources: Navigation Support to Access Resources

Family caregivers thought health providers should assist them to access the resources they needed to support their caregiving and to maintain their own wellbeing. Family caregivers of home care clients and congregate care residents reported a circular challenge of not knowing what might be available to help them and not knowing what they might need or could ask for. Family caregivers of congregate care residents had an additional challenge: they needed to understand how much they could be involved in residents’ care.

Family caregivers in all settings asserted that organizations were assembling ever longer lists of services and supports, yet what they needed was guidance about supports targeted to their needs. Several family caregivers suggested that what they needed was a navigator with whom they could have a conversation about their situation and who would then assist them to access the needed resources. Table 1 illustrates the perceptions of family caregivers from four care settings regarding factors related to navigation in the COVID-19 pandemic.

## 4. Discussion

Almost a year into the COVID-19 pandemic, the participants in this study thought that community and healthcare services should have been able to create a balance between protecting the care recipient and family caregivers from COVID-19 infection and preserving care recipients’ quality of life. In family caregivers’ views, the initial reaction to the pandemic was reasonable, but it took too long for healthcare institutions to respond to family caregivers’ needs. They charged that the COVID-19 pandemic exposed an under-resourced system of supports for family caregivers and care receivers in the community and in congregate care settings. The pandemic, along with moving services and supports online, exacerbated the silos within healthcare teams and within health systems. Family caregivers reported that often they were excluded from medical appointments or not asked about their knowledge of care-recipient in medical appointments. Communication and navigation were difficult. Thus, it was not surprising that participants wanted recognition of their caregiver roles as partners in care, partnering communication and navigation supports specific to their needs and goals.

All participants in this study considered the continuing care system (home care, supportive living, long-term care) to be under-resourced. Several family caregivers in supportive living and long-term care reported feeling they had to assist with residents’ care to ensure their residents’ quality of life was maintained. American caregiving scholars also emphasized that COVID-19 exacerbated existing weaknesses in support for family caregivers [49]. These interviews also affirmed our [14,37] and others’ [12,13,49] quantitative survey results that family caregivers’ care work, anxiety, and loneliness increased in the COVID-19 pandemic. To illustrate, Beach and colleagues [49] reported that COVID-19 “increased the effort involved in providing care, made it more physically, emotionally, and financially difficult, made it harder to get prescription medications, interfered with doctor appointments or treatment for the care recipient, and made it more difficult to obtain healthcare”.

The strict no-visitor policies that excluded family caregivers from long-term care centers received the most press attention [22] yet the family caregivers in all four settings reported negative impacts of program and service closures. While initially they respected restrictions to keep the care-receivers, they thought the health system have found a balance between restrictions for safety and services that would have supported their caregiving and the care-receiver’s and their wellbeing. The negative impacts of isolation caused by COVID restrictions on care-receivers’ [22,50,51] and caregivers’ [15,49,52,53,54] physical, mental, and cognitive health are now well documented. Family caregivers reported that the silos in healthcare, community, and government services that were present before the COVID-19 pandemic were exacerbated and complicated by closures, moving supports online, and staff working from home. Professional navigation models exist [55,56]; however, typically family caregivers are the “glue “coordinating care in siloed health systems [7,57,58]. In 2017, well before the COVID pandemic, Taylor and Quesnel-Vallée [59] estimated that family caregivers were spending 15–50% of their time trying to find and access services and supports. On a positive note, many family caregivers were pleased that they did not have to leave home for telehealth appointments and support sessions or family meetings on ZOOM. It would be useful for family caregivers, health providers, and researchers to work on strategies to coordinate care virtually

Study participants provided suggestions to improve family caregiver’s situations. They proposed that family caregivers should be recognized as partners in care, with the recognition of the caregiver role in policy and practice. These recommendations reinforce themes in research published before the COVID-19 pandemic [4,7,8,49] as well as expose the lack of progress in recognizing the family caregiver role and development of consistent family caregiver support. Recognition of the family caregiver role as integral to sustainability of the Canadian healthcare system was first raised in 2002 in the Future of Healthcare in Canada report [60]. In his retirement paper, “The Past is Prologue: Advancing Caregiver Interventions”, caregiving scholar Stephen Zarit [8] highlights the lack of progress in family caregiver support and recommends approaches that recognize family caregiver diversity and accommodate the changing needs of family caregivers over the caregiving trajectory.

Communication with family caregivers was deficient in the pandemic. In our July 2020 survey, only 32% of the family caregivers had been asked by a healthcare provider how they were or about caregiving [37]. Indeed, several interview participants began to cry when asked how they were doing in the interviews. Tears were followed by the explanation that no one cared about them. The International Alliance of Carer Organizations identified communication and information exchange between family caregivers and health providers as an area in need of improvement before the pandemic [61]. COVID-19 further impacted communication negatively [61,62]. Wittenberg et al.’s review of healthcare provider communication was challenged by reduced channels for communication with family, lack of time, and provider burnout [61].

Family caregiver’s ability to navigate the healthcare and social care systems suffered from the same pressures that affected communication. COVID-19 restrictions often limited family caregivers to searching for resources online. They wanted guidance from health and social care providers. As the pandemic continues, it is likely that family caregivers and providers will have to work out new communication and navigation strategies, many of which may be through telehealth or online [61,63].

The COVID-19 pandemic exacerbated the challenges of family caregiving [12,13]. The family caregivers participating in these interviews believed there should be tangible recognition of family caregivers in policy, improved communication with healthcare providers, and navigation support. Canadian policy makers currently agree that resources are needed to support seniors and family caregivers [64,65]. However, funding needs to be attached to policies to ensure sustained supports [66]. Several participants in this study thought that significant advocacy to ensure family caregiver’s needs remain on the policy agenda. We intend to mobilize the knowledge from this research to assist with this advocacy.

### Strengths and Limitations

This study themed family caregivers’ views across four care settings in the caregiver’s own home, the care-receiver’s home, supportive living, and long-term care. They represent the care trajectory, a relatively independent care recipient living in their own home who needs caring for a few hours a week for transportation or grocery shopping, a care recipient with complex care needs who can still be cared for at home and a resident of congregate care with care needs greater than family caregivers can provide with home care assistance. The healthcare system seems to assume that family caregiving stops when people are admitted to a group home, supportive living, or long-term care. For most family caregivers, caregiving is a long-term commitment that spans the entire care trajectory until the person they are caring for passes away [5,8].

A limitation of the present study is that participants were drawn from family caregivers who participated in an online survey of family caregivers. The family caregivers completing the survey were likely engaged caregivers with the skills to access and complete the survey on the Internet in English rather than under-resourced caregivers who might not have funds or literacy to access to the Internet. Family caregivers from other cultures with different norms beliefs and values may not have been able to access the survey. In the online survey and in these interviews, rural and urban family caregivers participated. Some rural participants noted that poor Internet service limited their participation in many of the online supports offered in the COVID-19 pandemic. The offer of interviews on either telephone or ZOOM made it easier for rural participants without reliable Internet to participate.

The uncertainty of the COVID-19 pandemic might have influenced family caregivers’ responses to the research questions. We began these interviews in January 2021 when vaccines were beginning to offer hope that public health protocols would ease. COVID-19 positivity rates were falling in February and March; however, as we were completing the last few interviews, Alberta was facing a third wave with the highest positivity rates in Canada and in North America. Despite the COVID-19 uncertainty, we found the themes remained consistent over the course of the interviews and the themes aligned with research conducted prior to the COVID-19 pandemic.

## 5. Conclusions

This study contributes new knowledge regarding family caregivers’ expectations for changes they would like in the future. Given the growth in the need for family caregivers and their contributions to the people they care for and to the healthcare system, it is critical that the family caregiver role be defined and then recognized in policy, funding, and practice.

## Figures and Tables

**Table 1:** Comments of family caregivers regarding factors related to the COVID-19 pandemic. Four care settings are represented.

Lives With	Lives Separately	Supportive Living	Long-Term Care
Family Caregivers’ Situations and Their Main Concerns			
Balance the Risk of COVID-19 With the Quality of Life.			
We had to weigh our chances of transmission of COVID or our mental health. So, a balance. During the day while my husband was at work, and I got respite so I could go grocery shopping or whatever. But now we’ve transferred that respite to us spending time because if we’re not solid as a couple then well, his care goes out the window. (Caregiver to immunocompromised child)	I’m not too worried, I think part of it was following the guidelines and I was still going in. But between my mom and I, we thought, if we do get it and if she dies or I die, it’s better to be together and have this a meaningful time together than to not see her and not know what’s going on behind the door. So, I’m feeling comfortable with that decision. (Caregiver to mother)	I think the biggest challenge is just the lack of social interaction, lack of family interaction. My mom has not been able to see her family in any real substantial way for a year. That’s my biggest concern. I was less concerned about COVID itself considering the level of screening and care that was being provided. That that was my biggest concern as well as the virus itself. (Caregiver to mother)	I have to say that [LTC] has had an amazing record of keeping cases to a minimum. So, they have done their job, certainly in terms of medical care and keeping residents safe. But I think the other aspects of the mental, social, and emotional support have suffered. Above all, they kept them safe, but they missed out on social relationships and real quality of life. (Caregiver to mother)
**What Could Have Been Done to Support Family Caregivers During the Pandemic?**			
Increase Resources to the Continuing Care System.			
They’re looking to cut as many costs as they can, but it doesn’t paint a very nice picture of what you get for care. The entire system is under-resourced. As we saw with the COVID, one of the big problems was the healthcare aides, who do a hell of a good job, had to cross over between private residences to acute care, designated supportive living residences, all of those because they couldn’t get a full-time job. (Caregiver to father)	Yes, the biggest problem with homecare is the constant changes of new people coming in and not feeling confident that they might bring COVID-19 in with them. And if they’re working at long term care facilities and then going to a personal home. That is not a new issue in home care. (Caregiver to mother)	With the two it is a heavy load. And I’m finding that my daughter’s group home has been fine, but where my husband is in supportive living, I need to spend more time there. Generally, like in all facilities, they’re short staffed, and so the three healthcare aides out of the five that do come in, and they’re run off their feet, especially seven to three shifts. (Caregiver to husband and daughter)	Yes, I personally think they’re underfunded from what I’ve seen. I used to go at least five days a week and spend from two to three hours a day with her. The more time I spent there, the less time staff felt that they had to spend with her. They’re so understaffed that when you can’t go visit now, all of a sudden, that resource is gone and now they’re hooped. (Caregiver to aunt)
Develop a Timelier Pandemic Plan.			
Another thing the [Gym] started to do, they were offering some online exercise classes, very similar to what we were getting off of the PBS channel on TV. But because it looked like they were going to open again in a month they kept waffling on opening and closing and they never really put the resources and time into it. The restrictions were going to lift or partially lift. So, you didn’t get the place open, and you didn’t get the proper online support. (Caregiver to wife)	Well, certainly the home care should have had some directives or some protocols back in March when the whole thing hit. Because they didn’t have anything at that time, I will say that a couple of months, maybe three or four months after I had taken over everything, that I did get a call from one of the Supervisors. But really, they should have been able to get their ducks in a row long before that. (Caregiver to mother)	I don’t understand it. We were locked out from March until July and they should have planned what to do. But they are still short staffed. And since COVID, it’s amazing how I can see deterioration in quite a few of the residents. (Caregiver to husband)	Well, I think that the facility itself should have looked at the people like my mom and what damage is done. There’s a distinct difference between somebody who’s visiting and somebody who’s actually a care provider. And was there a way to mitigate the risk of us continuing to do what we were doing to my mom’s benefit? Now, she’s at the stage of Alzheimer’s disease where there’s very little can be done with her other than that one-to-one personal contact. Person-centered was gone. (Caregiver to mother)
Reduce Silos in Healthcare.			
I couldn’t get rehab therapy because it’s out of the hospital. And they didn’t want people coming in that had already been discharged. Everything was just cut off that normally you could have gotten. And yet that’s so silly because at home, it’s just the two of you, it’s not as if you are going to have COVID-19. You’d be more likely to get it in the hospital from them. There’s been such protection around acute care and nothing for people at home. (Caregiver to husband)	It boggles my mind that, like all these programs that we’ve tried, I mean, we’ve really tried to get my aunt on some caregiving funded programs. It has been beyond a nightmare trying to help her in these situations because although the money may be there and in theory a program exists, that does not mean that a program is accessible and that the average person who is maybe not that skilled with these types of applications can actually access the funds. (Caregiver to grandmother)	Okay, my biggest challenge is there’s this huge discord in communication between the lodge and our home care and the doctors. And that communication is frustrating. Now, we have more home care coming into the lodge and doing more things, but in order for anything to happen with my mom, the Doctor has to do it through home care. He can’t do it directly to the lodge manager because she’s employed by home care. (Caregiver to mother)	Every time he has been moved, I have to learn the ropes all over again. We can’t keep our family doctor; they gave us a new one in [Facility 1] and then when they transferred him to [Facility 2] we were assigned another family doctor. He didn’t know the last family doctor; he doesn’t know the new family doctor and I don’t either. We don’t know the staff or how the system works. Some things are better though. I had to pay for his medications and incontinence supplies when he was in supportive living, but that is covered in long-term care. (Caregiver to husband)
**What Supports do Family Caregivers Think They Need in the Future Based on What They Learned in the COVID-19 Pandemic?**			
Recognition of the Family Caregiver Role.			
I agree that respect and support for the caregiver is underestimated part. When our case manager first met us and started to think she couldn’t believe that we were doing this 24 hours a day, 365 days a year, she immediately put in for the respite. But we never asked for it. I wish I’d known about that years earlier. (Caregiver to child)	I think there should be more recognition for the family caregivers and the value that they bring. I don’t think that caregiving is valued as much as it should be, and the current system is not working. I mean, there’s people that are in positions to help their family with caregiving, but we need a better partner. (Caregiver to mother)	And I think for caregivers all the responsibility for things the lodge can’t do now becomes yours. One person is the essential caregiver. So, whether it’s just visiting socially, her washing, taking her to appointments or picking up her grocery list, everything becomes your responsibility at some level. But no one says thanks. (Caregiver to mother)	Yeah. I mean, knowing that your concerns are heard is great. They need to recognize the caregivers. You want to know that you’re not being ignored. (Caregiver to mother)
Partnering with Communication Between Healthcare Providers and Family Caregivers.			
There needs to be better communication about what the home care staff can and need to do. Well, even the respite care, you have to show them the suction machine, some of them been trained but not all. Some wanted to call the office and have somebody come out and train them. And that stuff was in the folder when they came in the door, and they had to read what they’re here for. And once again, many overemployed so, undertrained for what they had to do. (Caregiver to wife)	Well, the communication needs to be improved. In business you can’t have people in top management positions coming up with all the ideas and making all the decisions. You need the front-line workers to be trained in communication. They need to tell management where the challenges are. You need to know from the families what we need, what could be better. And then you put a plan together. Everybody in this together. You really need it from a personal perspective, from front line care workers, families, clients. (Caregiver to mother)	There are certainly newsletters that come out very frequently in beautiful large type and well presented once or twice a week. So, they communicate well in a written perspective, but not in terms of direct one-on-one. Really there was no kind of conversation from the staff about my mother. (Caregiver to mother)No, communication is very poor. If we have concerns, we are to contact the LPN. And so, on several occasions, when that happens messages don’t get through. (Caregiver to father)	I find that there’s lots of information coming out of the facility in the form of emails and that sort of keeps you up to date. The one thing I do find is that the meetings are less of a discussion and more about here’s what we’re doing and if you have any concerns. Well, a lot of people who don’t have my background don’t know what to be concerned about. So, there’s not a lot of concern expressed because families don’t even know what questions to ask. (Caregiver to mother)
Access to resources: Navigation support to access resources.			
And I certainly hope that those people that need the help get it, and the one thing I guess that really irks me is the fact it’s so difficult to find out about these programs. It takes years to know about them. You know, even Caregivers Alberta, I only just found out about this past year, and I’ve been a caregiver for my mom since 2007. Here’s the rub. I can find out more about my car on the web than I could ever find out about what I needed as a caregiver. (Caregiver to father)	If I had half an hour extra day, I would go online and research what potential things I could use. But I don’t have time. By the time I make it home at night, I have dealt with crises all day. I’ve got nothing left, just nothing. And I’m a researcher. I’ve developed programs. By the end of the day, I can barely move. So, who has time to get on the computer? (Caregiver to mother and father)	I don’t think those finer grained, how things work is explained. From my experience, it is better to work from the bottom up to get what [name] wants, then it is to work from the top down. I talk to his immediate caregivers. They look after the things that [name] and I want looked after. But no, what families can do and can’t do is not clear. You have to learn it bit by bit. (Caregiver to husband)	Does anyone really understand appropriate programs and services across Alberta’s continuing care levels? A key system gap is ensuring engagement of caregivers given the level of the care continuum. They come into the system not knowing what services are provided at that level, what they need to do, or must arrange to have done. Really families need a LTC receptionist that will bridge the information sharing gap. Someone to explain the system and how to navigate it. (Caregiver to mother)

## Data Availability

Data are available from the authors on request. Email sdanders@ualberta.ca.

## Supplementary Materials

The following are available on https://www.mdpi.com/article/10.3390/diseases9040070/s1. Supplementary Material: Family Caregiver Interview Guide; Table S1: Table stages of thematic analysis.
