# Supplementary material for: Neglected Needs of Family Caregivers during the COVID-19 Pandemic and What They Need Now: A Qualitative Study
Source: Diseases. 2021 Oct 13;9(4):70. doi: 10.3390/diseases9040070 (PMC8544374; doi:10.3390/diseases9040070)
Supplement: Supplementary file 1 [file diseases-09-00070-s001.zip › diseases-1380746-supplementary.pdf]

Supplementary Material: Family Caregiver Interview Guide

Family Caregiver Interview Guide

Overview:

The purpose of the interview today is to hear from you how providing care has changed during the pandemic, what you think could have been done to support you and the person that you care for during the pandemic and then what you think you need as the COVID-19 is controlled.

First, we have ethics for your verbal consent to participate in this interview. Have you read the information letter about the study that I sent by email? Will you give verbal consent to participate in the interview?

Thank you. Your point of view as a family caregiver is important. You can answer as many or as few questions as you would like. If you don't want to answer a question, just let me know and we can move on. Being mindful of your time, this should take 45 minutes to 1 hour. Again, we can stop at any time. May I record the discussion to facilitate recollection and accurate reporting? (If yes, switch on the recorder).

Anonymity:

Despite being recorded, I would like to assure you that the discussion will be anonymous. The recordings will be kept for 5 years as per university policy in an encrypted folder on a secure university server. The recordings will be transcribed word for word. The transcribed interviews will be "cleaned" so that information that would allow individual subjects to identified will be removed. The transcribed interviews will be stored in a separate folder from the recordings.

Guiding questions:

1. First, can you tell us little bit about their caregiving situation, if you feel comfortable.

   Probes:
   - How has your caregiving changed during the COVID-19 pandemic?
   - What has been your biggest worry during the COVID-19 pandemic?

2. What do you think should have been done differently?

   Probes
   - What resources you have benefitted from?
   - What might have made your life/ caregiving easier during the pandemic?

3. What could have been done to support you and the person you care for during the pandemic

   Probes:
   - Have you experienced any relief or positive experiences during the COVID-19 pandemic?
   - Did you experience any difficulties in coordinating care because of COVID-19 restrictions?
   - Has anyone asked you about your wellbeing and your caregiving?

4. Now, as the pandemic ends and things become more normal, what do you think you need?

   Probes:
   - What resources might help you to care?
   - What might make it easier for you to care?

- What might help you to maintain your own health?

Concluding question:

5. Now I have asked you lots of questions, what do you think we should know about?

Conclusion:

Thank you for participating. Your experiences and recommendations are really important for us to help policy makers and the public aware of what family caregivers do and what they need. If there is anything you are unhappy with or wish to complain about, please contact me or you can call the Chair, Conjoint Health Research Ethics Board, University of Alberta. The number is on the information sheet.

If you think of anything else that you would like to tell us, you have my email address and cell phone number. Thank you again

Table S1. Table stages of thematic analysis.

Step 1	Researchers familiarize themselves with data	Read transcripts and listen to the recordings. Make corrections in transcriptions, Make notes on tone, first impressions of the data.
Step 2	Identify preliminary codes	Identify interesting elements in data.<br>Document impressions with notes.<br>Write memos on emerging themes.<br>Document connected elements.
Step 3	Find themes in the data	Look for and identify themes in the data.<br>Identify all data relevant to the themes.
Step 4	Finalize the themes	Finalize the themes.<br>Check for data overlaps between the themes.
Step 5	Review each theme	Review the data to ensure that data fits each unique theme. Reread the transcriptions to ensure the overall data fits with the overall storyline and each theme.
Sept 6	Document and review documented analysis	Analyze the resultant documentation and the inferences drawn.
